# Efficacy and indication optimization of Chinese medicine (*Tiao-Chang Ke-Min* granules) for diarrhea-predominant irritable bowel syndrome: study protocol for a randomized controlled trial

**DOI:** 10.1186/s13063-018-2754-9

**Published:** 2018-07-11

**Authors:** Qian Li, Bei-ping Zhang, Shao-gang Huang, Wen-wei Ouyang, Jian-hui Xie, Ze-huai Wen, Xiao-bo Yang

**Affiliations:** 1 Chinese Medicine Syndrome Research Team, Guangdong Provincial Hospital of Chinese Medicine, No. 111, DaDe Road, Guangzhou, 510120 China; 2 The Second Clinical College of Guangzhou University of Chinese Medicine, No. 12, JiChang Road, Guangzhou, 510405 China; 3 Department of Gastroenterology, Guangdong Provincial Hospital of Chinese Medicine, No. 111, DaDe Road, Guangzhou, 510120 China; 4 Key Unit of Methodology in Clinical Research, Guangdong Provincial Hospital of Chinese Medicine, No. 111, Dade Road, Guangzhou, 510120 China

**Keywords:** Irritable bowel syndrome, Diarrhea-predominant, Randomized controlled trial, Prediction of the treatment outcome, Indication, *Tiao-Chang Ke-Min* granules, Chinese medicine

## Abstract

**Background:**

Irritable bowel syndrome (IBS) is a chronic, recurring condition, prevalent in the general population. Current medication treatments usually leave patients undertreated. Nowadays, Chinese medicine (CM) is being considered as a promising treatment approach for IBS. However, due to methodological limitations, there is no strong evidence to support CM. Although IBS relapses are common, the relapse assessment has always been neglected in CM study designs. Meanwhile, in clinical practice and studies, it has been found that certain CM formulas can only benefit certain kinds of patients. Discovering what population and illness characteristics likely respond to outcomes may help improve the effectiveness of CM. The aims of this study are to evaluate the efficacy and safety of *Tiao-Chang Ke-Min* (TCKM) granules for IBS, especially in reducing IBS symptoms’ relapse, by a high-quality randomized controlled trial and then to optimize the indication of the TCKM granules.

**Methods/design:**

This is a parallel-group, randomized, double-blind, placebo-controlled trial embedded with outcome predictive factors. Eligible patients with diarrhea-predominant IBS will be randomized into either a TCKM granule group or a placebo group. Patients from both groups will receive health education. The treatment duration is 4 weeks and the follow-up is 12 weeks. The primary outcome is global improvement measured with adequate relief (AR). The second outcome measures include time until relief, time until first relapse, total relapse times, long-term effectiveness, individual symptoms, IBS-Symptom Severity Score (IBS-SSS), IBS-Quality of Life Questionnaire (IBS-QOL), and Hospital Anxiety and Depression Scale (HADS). Predictive factors associated with patient and illness characteristics have been widely collected. These factors will be embedded in this trial for further identification.

**Discussion:**

This trial may provide high-quality evidence on the efficacy and safety of TCKM granules for IBS and a more accurate indication. Importantly, this trial will provide a new research method for improving the therapeutic effects of CM for clinicians and researchers. To address IBS relapse assessment, a series of special definitions of relapse incidents has been made for this trial.

**Trial registration:**

Chinese Clinical Trial Registry, ID: ChiCTR-IOR-17010600. Registered on 9 February 2017.

**Electronic supplementary material:**

The online version of this article (10.1186/s13063-018-2754-9) contains supplementary material, which is available to authorized users.

## Background

Irritable bowel syndrome (IBS) is a chronic, recurrent, and functional gastrointestinal disorder. It is very common, affecting between 5 and 20% of the general population worldwide [1, 2] and 4.6–6% of the population in China [3, 4]. IBS is a heterogeneous disorder with a complex pathogenesis, making treatment challenging [5]. There has been no consensus on the specific and effective strategies for the treatment of IBS. Current medication treatments are mainly based on relieving abdominal symptoms [6]. Therefore, many IBS patients remain undertreated or dissatisfied with their quality of life [7]. In addition, the long-term effectiveness of treatments is not good enough, as a systematic review concluded, through long-term follow-up, that only about 30% of IBS patients’ symptoms improved or disappeared [8]. Recently, more and more IBS patients have sought to treat their symptoms with complementary and alternative medicine [9].

At present, Chinese medicine (CM) is considered a promising approach in the treatment of IBS. CM is, by nature, multi-targeted. It can manage IBS through simultaneously acting on diversified pathways and mechanisms [10]. Clinical trials of CM for IBS have shown that CM could provide effective improvement in single symptoms, global assessments, and quality of life with few adverse events reported [11, 12]. However, according to current reviews, due to a lack of rigorous larger clinical trials, there is no strong evidence supporting CM in the treatment of IBS [7, 13, 14]. Furthermore, although IBS relapses are common, relapse incident assessment has always been neglected in CM study design [15].

One thing related to the effectiveness of CM should be given particular attention. CM treatment is a system of individualized medicine. The development of a CM formula is specific for a group of patients who share the common characterizes based on CM theory. In clinical practice and studies, it has been found that CM formulas may lead to a benefit improvement in some of the patients, but not in all. Finding out what patient and illness characteristics likely respond to CM treatment may help to identify a more suitable patient population, and improve the effectiveness of CM [16]. Similar studies have been conducted in psychological therapy for IBS [17–19]. These studies identified factors predicting a good or poor outcome, which was expected to provide helpful suggestions for clinicians. However, until now, there have been no studies looking specifically at predictive factors of treatment outcome in CM trials for IBS.

*Tiao-Chang Ke-Min* (TCKM) granules were developed by Luo Yunjian, a famous practitioner of CM in China. They can play a crucial role in addressing the core pathogenesis and causes of IBS, according to CM theory. TCKM granules have an effect on reducing IBS symptom relapse, and its effectiveness and safety have been confirmed through long-term clinical practice. To date, however, there have been no high-quality studies conducted to test its effectiveness.

We plan to perform a randomized controlled trial embedded with comprehensive outcome predictive factors, for the purpose of establishing high-level evidence and setting an example for optimizing the indication of IBS treated with CM.

### Trial purpose

The specific objectives of this trial are as follows:

1. To assess the efficacy and safety of TCKM granules for IBS, especially in reducing IBS symptom relapse
2. To investigate what patient and illness characteristics may predict a successful outcome from the treatment of TCKM granules, and optimize the indication.

## Methods/design

### Design

**Fig. 1:**
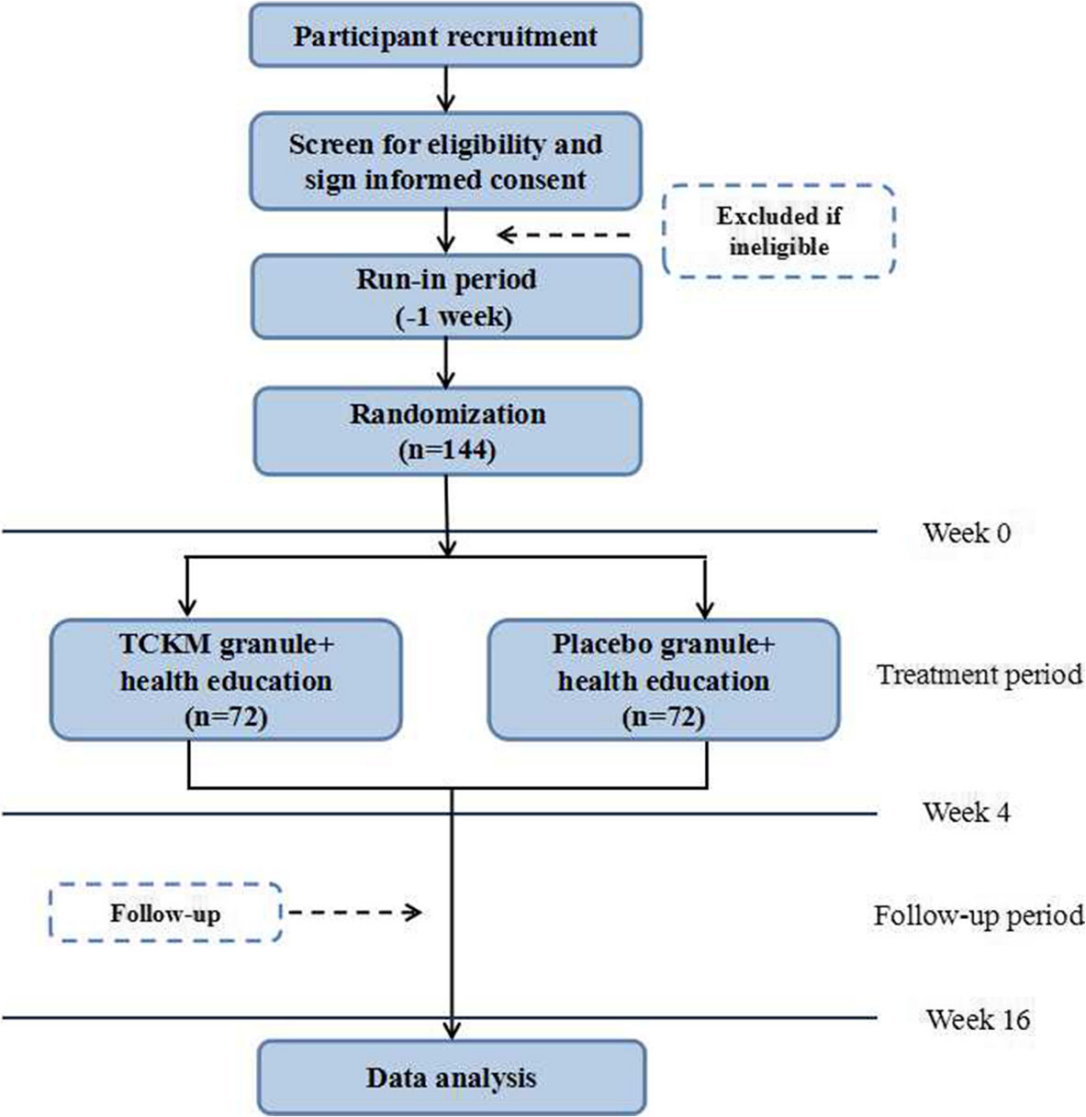
Flowchart of study. *TCKM Tiao-Chang Ke-Min*

**Fig. 2:**
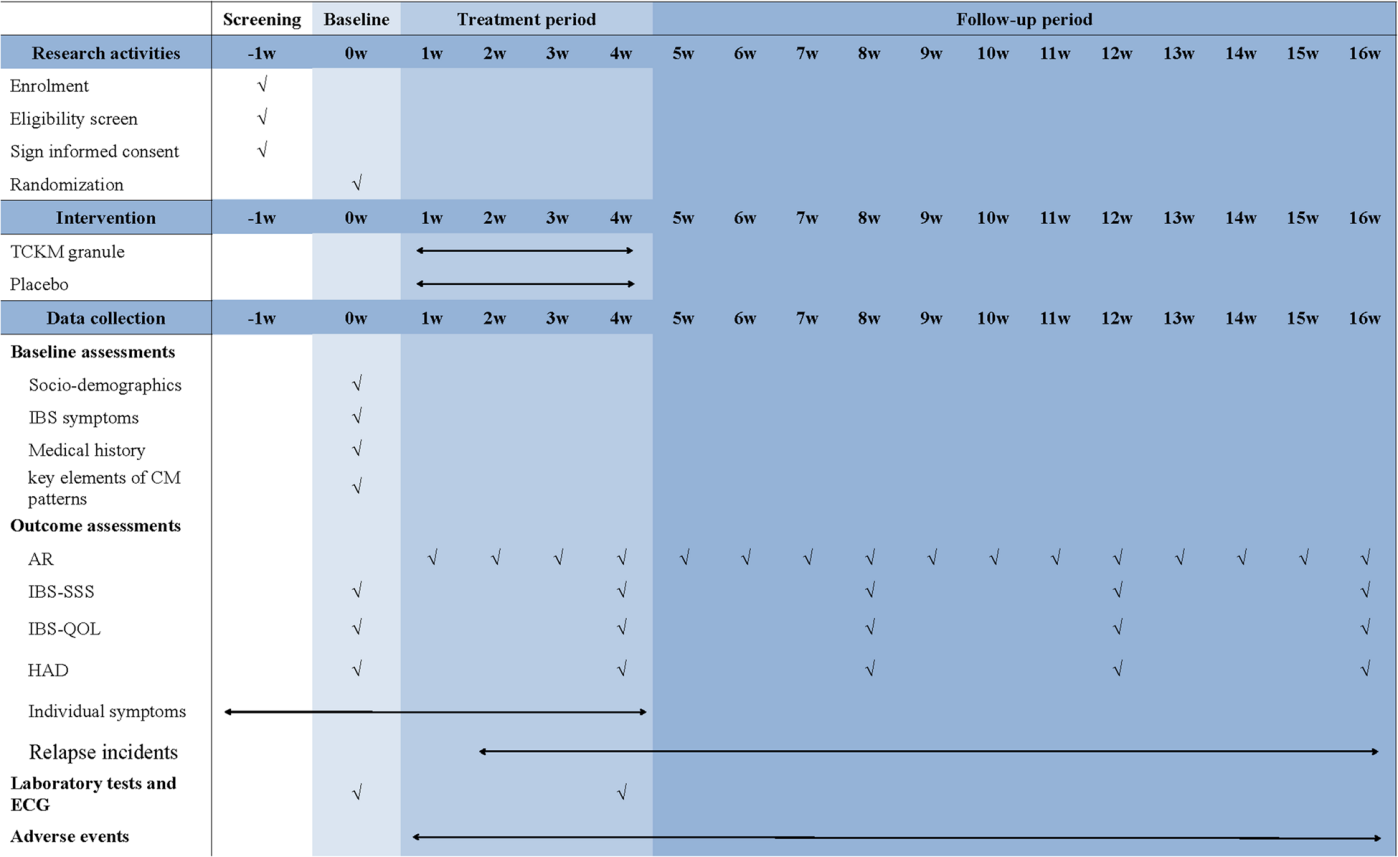
Study schematic diagram. *TCKM Tiao-Chang Ke-Min*, *IBS* irritable bowel syndrome, *AR* adequate relief, *IBS-SSS* IBS Symptom Severity Score, *IBS-QOL* IBS-Quality of Life Questionnaire, *HADS* Hospital Anxiety and Depression Scale, *AE* adverse event, *ECG* electrocardiogram

This is a parallel-group, double-blind, randomized, placebo-controlled trial. The trial will include three periods: a 1-week run-in period, a 4-week treatment period, and a 12-week follow-up period. During the run-in period, patients who fulfill the screening criteria will record a symptom diary every day, and stop taking any drugs that the trial forbids. Those, who record all items in the symptom diary for at least 5 days during the run-in period and remain eligible will be randomized at a ratio of 1:1 into either the TCKM granule group or the placebo group. Visits will be scheduled at weeks 0–16 (or at discontinuation) to assess treatment efficacy, recurrence of IBS symptoms, and occurrence of adverse events. Additionally, comprehensive predictive factors associated with patient and illness characteristics have been collected through a series of qualitative methods. These factors will be embedded in this trial for further identification. (See Figs. 1 and 2). The protocol design is based on the Standard Protocol Items: Recommendations for Interventional Trials (SPIRIT) Checklist (Additional file 1).

### Participants

#### Setting and recruitment

The trial will be conducted at three branches of the Guangdong Provincial Hospital of Chinese Medicine (GPHCM), Guangzhou, China: (1) Dade Road Hospital, (2) Ersha Island Hospital, and (3) Guangzhou University City Hospital. The recruitment of participants for this trial will be mainly via physician referrals from the gastroenterology clinics. Advertisements and colored fliers will also be placed inside the hospitals. Those who are willing to participant in this trial will need to contact the research assistants by telephone.

#### Diagnostic criteria

Diagnosis of IBS must meet the ROME IV criteria [20]. Among IBS subtypes, participants will be included in the type of diarrhea-predominant IBS. However, CM patterns of patients will not be restricted.

#### Inclusion criteria

1. Diagnosis with diarrhea-predominant IBS
2. 18 to 75 years of age, male or female
3. Signed informed consent
4. A reported Irritable Bowel Syndromes Symptom Severity Score (IBS-SSS) of > 75

#### Exclusion criteria

1. Patients with alarm symptoms such as severe weight loss in the past few months and hematochezia
2. Patients with history or current evidence of inflammatory bowel disease
3. Patients with history or current evidence of celiac disease, concurrent enteric infection, hyperthyroidism or other diseases causing diarrhea and abdominal pain
4. Patients who report a score of > 50 on the Self-rating Anxiety Scale (SAS) or a score of > 53 on the Self-rating Depression Scale (SDS), or patients with other psychiatric disorders
5. Patients with a history of gastrointestinal surgery causing ailments similar to IBS
6. Patients who are unable or unwilling to stop taking medication affecting the assessment of experimental drug efficacy and safety
7. Patients who are pregnant, breastfeeding, or plan to conceive within 3 months
8. Patients with concurrent serious diseases, such as cancer, serious cardiovascular, respiratory, hepatic, renal, or neurological disease
9. Patients who are participating or who have participated in another clinical trial within the previous 3 months
10. Patients who, in the opinion of the researcher, are unsuitable for the trial

### Randomization

A block randomization sequence will be created by an independent statistician, using SAS 9.2 software (SAS Institute Inc., Cary, USA). The randomization will be stratified by site with eligible participants randomly assigned to either the TCKM granule group or the placebo group at a ratio of 1:1. Researchers will access the treatment allocation for each eligible participant through a remote and web-based randomization system, which has been developed by the Key Unit of Methodology in Clinical Research, GPHCM. A label with a serial number based on the randomization schedule will be attached to all drugs. Treatment allocations will be kept in password-protected files and saved by an independent staff member.

### Blinding

Treatment allocations will be blinded to the participants, clinicians, research assistants, drug managers, statisticians, and other staff members, and will not be revealed until the study is completed. After a run-in period, the clinicians will assess whether the patients are still eligible. For each eligible patient, the clinician will apply for a randomized assignment by logging into the web-based randomization system, and write a prescription for “TCKM granules, three bags, twice a day.” Then, accompanied with a research assistant, the patients will be taken to the appointed drug managers at the GPHCM pharmacy. The drug managers are responsible for dispensing medication according to the prescriptions and assignments. During the trial periods, clinicians, research assistants, and drug managers are forbidden from discussing the assignment possibilities with the participants. The placebo granules have an appearance, smell and taste similar to the TCKM granules. All drugs are packaged in an identical manner. The blinding codes will be kept strictly confidential, and will not be broken during the trial unless serious adverse events occur. The date and reason for breaking the blinding code will be recorded in the case report forms (CRFs).

### Intervention

1. Health education. The participants in both groups will receive a health education course. Health education handbooks will be provided by research assistants, and will include a series of IBS-related information, such as pathophysiological mechanisms, causative factors, and prognosis. After reading their handbooks, the participants can discuss the material with the clinicians. Diet- and stress-related issues among IBS patients will be discussed in details
2. Experimental drugs and placebo. Participants will receive three bags of either the TCKM granules or the matched placebo granules twice a day for 4 weeks. The placebo consists primarily of lactose, maltodextrin, natural food coloring and sucrose octaacetate. The experimental granules and the placebo granules are identical in appearance, taste, smell, weight, and packaging. Both of them will be provided by the Tianjiang Pharmaceutical Co., Ltd. (Jiangyin, Jiangsu Province, China) which meets the requirements of the Good Manufacturing Practice (GMP). All herbs will be obtained from qualified suppliers in China
3. Rescue medication. If patents’ diarrhea and abdominal pain are exacerbated beyond a tolerable level, rescue medication will be allowed for relieving abdominal symptoms in both groups. Montmorillonite powder, berberine hydrochloride, and levofloxacin will be allowed for uncontrollable diarrhea; and pinaverium bromide will be allowed for uncontrollable abdominal pain. Concomitant use of two or more rescue medications will also be allowed, and their dosages will depend on the symptoms severity. However, the administration of the rescue medication must be limited within 3 days, or the patients will be considered as withdrawn. In such cases, the name, dosage, and duration of the rescue medication are to be documented in the symptom diaries and CRFs

### Outcome assessments

#### Primary outcome

The primary outcome of this trial is adequate relief (AR), which is usually used to evaluate IBS global symptom improvement [21]. The participants will be asked the following question at each visit during the treatment period: “In the past 7 days, have you had adequate relief of your irritable bowel syndrome pain and discomfort?” Participants will be considered as responders when they answer “yes” at least twice during the 4-week treatment period [22]. Only a follow-up visit and a single positive response will be considered as non-responders.

#### Secondary outcomes

##### Relief and relapse incidents

The definition of relapse is the recurrence of abdominal pain/discomfort or diarrhea in IBS patients who have achieved treatment success. Achieving treatment success means that IBS symptoms have been relieved, and the relief has lasted at least 1 week after treatment. Relief and relapse incident outcomes include time until relief, time until first relapse, and total relapse times. Time until relief is defined as the time from patients receiving treatment to achieving treatment success. Time until first relapse is defined as the time from patients achieving treatment success to the recurrence of abdominal pain/discomfort or diarrhea. Total relapse times refer to the sum of relapse times during both the treatment period and the follow-up period.

##### Long-term effectiveness

Participants who will be weekly AR responders for at least six of the 12 weeks during the follow-up period will be considered long-term effectiveness responders [23].

##### Individual symptoms

Throughout the whole study period, patients must record their IBS symptoms on paper diary cards every night before going to sleep. Diaries recorded in the run-in and treatment periods will be used to assess the efficacy and safety of the treatment, while diaries recorded during the follow-up period will be used to avoid potential bias from symptom recall. Individual symptoms are to be recorded as follows: severity of abdominal pain, abdominal discomfort, and abdominal distension evaluated by a 5-point Likert scale (0 = none; 1 = mild; 2 = moderate; 3 = severe; and 4 = intolerable); stool frequency; stool consistency classified by using the Bristol Stool Form Scale (BSFS) [24]; feelings of incomplete evacuation, urgency, and mucus evaluated on a binary scale (0 = absent; 1 = present).

##### Other outcomes

Other outcomes include the scores of the Irritable Bowel Syndromes Symptom Severity Score (IBS-SSS), Irritable Bowel Syndrome-Quality of Life Questionnaire (IBS-QOL), and Hospital Anxiety and Depression Scale (HADS) [25–27]. The questionnaire and scales above will be completed at the run-in period baseline (week 0), the end of the treatment period (week 4), and during the follow-up period (weeks 8, 12, and 16).

### Safety assessments

All participants must undergo an electrocardiogram examination and a series of clinical laboratory tests before and after the treatment (weeks 0 and 4). These will include routine blood, urine, stool, and stool occult blood, kidney function, and liver function tests. More attention will be given to the abnormal changes found in the examinations and, when necessary, a re-examination and a causality assessment will be required.

No reports of harm to patients exist from long-term clinical observations with TCKM granule treatment. Nevertheless, participants will still be instructed to report any adverse events (AEs) during the trial. The clinicians will be in charge of managing AEs. An independent Data and Safety Monitoring Board (DSMB) from GPHCM will be in charge of studying each case individually to access any serious adverse event (SAE) and the causality between the SAE and the intervention. AE details including clinical manifestations, severity, occurrence time, recovery time, management and causality will be recorded on CRFs. If any SAE occurs, it must be reported to the principal investigator, DSMB, and the Ethics Committee within 24 h, and any necessary treatment will be provided as soon as possible. When necessary, the blinding will be broken and participants will be provided suitable treatments. All SAEs will be followed up until they have been resolved.

### Data management and quality assurance

Before the conduction of the clinical trial, all investigators and relevant staff including clinicians, research assistants, and drug managers will be uniformly trained regarding the trial-specific process. The training will be conducted by the principal investigator, and a study protocol and standard operation procedure documents will be provided. This will ensure consistency among researchers undertaking the same tasks, and ensure that their actions remain in strict accordance with the study protocol. Data collection for this trial will be performed by investigators and research assistants. All collected data will be checked regularly by the data management personnel, and be overseen by monitors from the Guangdong International Clinical Research Center of Chinese Medicine (Guangzhou, China). Double data entry of CRFs will be conducted by two experienced independent data entry clerks. At the time of analysis, outliers in the resulting spreadsheets will be checked to eliminate errors in data entry. All information collected in this trial will be kept in strict confidence, and only authorized staff will have access to the trial data.

Considering that those IBS patients accompanied with psychological symptoms are more likely to withdraw, measures will be taken to facilitate maximization of their compliance. For example, before every visit, a research assistant will contact the patient to confirm the schedule; specialized telephones for this trial will be installed, in order for patients to conveniently contact clinicians or other staff. However, if patients withdraw from the trial, outcome assessment, the last date of patients taking medication, and the reasons for withdrawal will be recorded as soon as possible. We will also take some measures to monitor the drug intake by participants. For example, the participants will be required to record a symptom diary and answer the question: “Did you take the *Tiao-Chang Ke-Min granules* today?” before going to bed every day. Besides, the research assistants will ask the participants “How many packages of the granules have you taken in the past week?” weekly during the treatment period and complete the CRF.

### Sample size calculation

The sample size for this trial was calculated based on the primary outcome—the proportion of responders of AR, using PASS 11.0 software (NCSS, LLC, Kaysville, UT, USA). Combining our previous observational study with similar studies [28, 29], we assume that the proportion of responders of AR in TCKM granule group will be 60%, and in the placebo group 30%. Fifty-seven patients in each group can achieve 90% power and rule out type I error of 5% by using a two-sided test. We expect that 20% of the participants may be lost to follow-up. Therefore, the sample size in each group should be adjusted to 72. In total, 144 participants will be enrolled in this trial.

### Design of outcome predictive factors

Outcome predictive factors have been investigated by a series of qualitative methods, including academic literature research, questionnaire survey, and expert consensus conference. Predictive factors were collected mainly around three cores and four levels. The three cores refer to causative and risk factors of an illness, state of an illness, and different classification conventions from both Western medicine and CM. The four levels include demographic characteristics, clinical characteristics, systems biology characteristics, and classification assessments along multiple dimensions. These factors will be embedded in this trial for further observation and analysis. Factors that may predict successful outcome from the treatment will be selected.

### Statistical analysis

Statistical analysis will be performed by blinded specialized statisticians using PASW Statistics 18.0 (IBM SPSS Inc., Armonk, NY, USA) and SAS 9.2 software (SAS Institute Inc., Cary, NC, USA). A level of 5% (two-sided) type I error will be considered as statistically significant. Analysis will be based on both intention-to-treat and per-protocol populations.

Baseline characteristics, which also include predictive factors, will be presented for each group. From baseline to each time point, discrete variables will be described with frequencies and percentages, and continuous variables will be described with either mean and standard deviation for data with normal distribution or median and interquartile range for non-normally distributed data. For comparisons between experiment and control group at each time point, a chi-square test or Fisher’s exact test will be used for discrete variables, and the Student’s *t* test or Wilcoxon-Mann-Whitney *U* test will be used for continuous variables.

The analyses of predictive factors will be performed in two steps as follows: The first step is univariate analysis. The AR post-treatment (week 4) and follow-up (week 16) responder rates will be used as dependent variables, and predict factors, such as demographic and clinical characteristics, and key elements of CM patterns, will be used as independent variables. A logistic regression analysis will be conducted. The selection criteria for the independent variable is defined as α = 0.1. The second step is multivariate analysis. Those predictive factors selected through the first step will be entered into two multiple regression models, taking the post-treatment (week 4) and the follow-up (week 16) AR responder rates as the dependent variable. Each model will be conducted to adjust or unadjust for initial baseline level including scores of IBS-SSS, IBS-QOL, and HADS.

We plan to do some sensitivity analyses. Firstly, we will compare the primary and safety outcomes between all randomized patients and unmasking patients. Secondly, we will test the impact of the missing data on our primary outcome. The method of multiple imputations will be used to handle missing data. We will compare the results of primary outcome using the multiple imputed dataset and un-imputed dataset to test robustness.

Planned subgroup analyses will be carried out in relation to the primary outcome—AR. The main analysis for each subgroup will be an unadjusted test of interaction in a logistic model.

The following status at baseline will be applied for subgroup analyses:

1. Demographic characteristics
2. Disease characteristics
3. Clinical symptoms
4. Key elements of CM patterns

### Protocol amendments

A formal amendment to the protocol will be required, and must be approved by the principal investigator and the Ethics Committee of the GPHCM prior to implementation, if modifications may affect the conduct of the study, patients’ potential benefit or safety.

## Discussion

As far as we know, this will be the first study to characterize the responders of treatment outcome along multiple dimensions for IBS in the CM field. This will also be the first study to focus on evaluating the recurrence of IBS symptoms with a series of special definitions of relapse incidents. Compared to previous studies of CM for IBS, this study will have more rigorous methodology and quality assurance.

Relapse is frequent among IBS patients, often leading to repeated clinical visits and missed work. Previous studies have usually been interested in evaluating the short-term effectiveness of an intervention, while recently more attention has been transferred to long-term effectiveness [30]. We have yet to find special outcome on the assessment of IBS relapse. Most studies have evaluated long-term effectiveness by repeatedly measuring common outcomes at each visit. However, the variation in conditions between two visits often is not readily available. Based on clinical practice, we make a series of special definitions of relapse incidents, including time until relief, time until first relapse, and total relapse times. These may be more appropriate for relapse assessment.

Some studies have suggested that in order to achieve a better understanding of the variation in patients’ responses to treatment effects, study populations should be described along multiple dimensions [31]. Doing this may also help to identify more suitable patient populations and optimize the indication. Current studies that have investigated predictive factors have usually been interested in gathering conventional variables such as demographic, clinical, and psychological characteristics [32]. Meanwhile, several potential important factors, such as biological characteristics and environmental factors, have been ignored [33]. Compared with previous studies, our study collects predictive factors along the multidimensional nature of IBS. In addition, due to the specificity of CM’s theoretical system, factors associated with CM, including the CM pathogenesis and symptom patterns, are also taken into account in this trial.

According to the CM theory, *Gan-qi* stagnation and *Pi-qi* deficiency are the basic pathogenesis for IBS [34, 35]; dietary intolerance and emotional disorders are two main causes, making IBS symptoms occur or recur [15]. TCKM granules are comprised of herbal medicine with the actions of regulating the function of the *Gan* and *Pi*, and resisting impacts of dietary and emotional factors. Therefore, TCKM granules could reduce IBS symptom relapse.

The results of this trial should provide high-quality evidence on the efficacy and safety of TCKM granules for IBS and more accurate indication of TCKM granules. Importantly, this trial will provide a novel research method for clinicians and researchers to improve the effectiveness of CM.

## Trial status

Participant recruitment is in preparation.

## Additional file

Additional file 1: Standard Protocol Items: Recommendations for Interventional Trial (SPIRIT) 2013 Checklist: recommended items to address in a clinical trial protocol and related documents*. (DOC 119 kb)

## Abbreviations

AE: Adverse event
DSMB: Data and Safety Monitoring Board
AR: Adequate relief
BSFS: Bristol Stool Form Scale
CM: Chinese Medicine
CRFs: Case report forms
GMP: Good Manufacturing Practice
GPHCM: Guangdong Provincial Hospital of Chinese Medicine
HADS: Hospital Anxiety and Depression Scale
IBS: Irritable bowel syndrome
IBS-QOL: IBS-Quality of Life Questionnaire
IBS-SSS: IBS Symptom Severity Score
SAE: Severity adverse event
SAS: Self-rating Anxiety Scale
SDS: Self-rating Depression Scale
TCKM: *Tiao-Chang-Ke-Min*
